# Reversible temperature-dependent high- to low-spin transition in the heme Fe–Cu binuclear center of cytochrome *ba*_3_ oxidase

**DOI:** 10.1039/c8ra09954e

**Published:** 2019-02-06

**Authors:** Antonis Nicolaides, Tewfik Soulimane, Constantinos Varotsis

**Affiliations:** Cyprus University of Technology, Department of Environmental Science and Technology P. O. Box 50329 3603 Lemesos Cyprus c.varotsis@cut.ac.cy; Chemical and Environmental Science Department, Materials & Surface Science Institute, University of Limerick Limerick Ireland

## Abstract

A reversible temperature-dependent high-spin to low-spin transition with *T*_1/2_ = −60 °C has been observed in the resonance Raman spectra of the equilibrium reduced and photoreduced heme *a*_3_ of the thermophilic *ba*_3_ heme–copper oxidoreductase. The transition is based on the frequency shifts of the spin-state marker bands *ν*_2_ (C_b_C_b_) and *ν*_10_ (C_a_C_m_) and is attributed to the displacement of the heme iron along the heme normal as a consequence of the Fe–Np repulsion at temperature below −40 °C which will increase the ligand field strength forcing the pairing of d electrons into the lower energy orbitals.

## Abstract 2

A reversible temperature-dependent high- to low-spin transition with *T*_1/2_ = −60 °C has been observed in the resonance Raman spectra of the equilibrium reduced and photoreduced heme *a*_3_ of the thermophilic *ba*_3_ heme–copper oxidoreductase.

## Graphical abstract

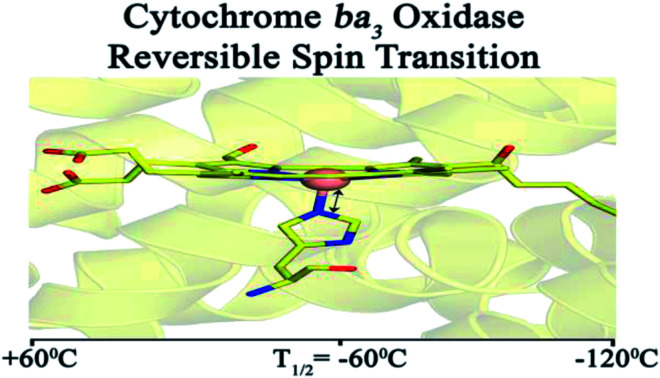

## Introduction

A variety of organisms perform their operations under extreme environmental conditions, such as high pressures and temperatures, high salt and non-physiological pHs.^1–3^ An adaptation mechanisms is a necessity by nature to stabilize the proteins from these organisms.^1–5^ Identifying the determinant factors which contribute towards the stability of the proteins is of profound importance due to importance of the physicochemical principles behind protein folding, stability, structure and function at high temperatures. The *ba*_3_-heme–copper oxidoreductase from the Gram-negative thermophilic eubacterium *Thermus thermophilus* catalyzes the reductions of O_2_ to H_2_O and of NO to N_2_O and also the oxidation of CO to CO_2_.^1–8^ The enzyme contains a binuclear center that consists of Cu_B_ and a high-spin (HS) heme *a*_3_ in which the Fe atom is in the plane of the heme and the distance of the heme *a*_3_ Fe to the proximal histidine ligand His384 is 3.3 Å (Fe–N_ε_). In addition, the distance from N_δ_ of His384 to the carbonyl of Gly359 is within H-bonding distance (3.0 Å), and the distance of N_ε_ of H384 to Asn366 is 3.3 Å. It also contains a homodinuclear copper complex (Cu_A_) and one low-spin (LS), 6C heme *b* which are part of the redox centers involved in the electron transfer processes for the catalytic activities of the enzyme.^1^

In *ba*_3_, the variation in protonation state of the *a*_3_ proximal heme Fe–His384 with Gly359 was invoked to account for the occurrence of the split Fe–His stretching mode, which has components at 193 and 210 cm^−1^.^9,10^ The conformer with the weaker (or absent) H-bond is expected to have the weaker Fe–His bond and the lower frequency vibration at 193 cm^−1^. The more strongly H-bonded conformer contributes to the 210 cm^−1^. It has been reported that the loss of intensity of the heme Fe–His384 mode at 193 cm^−1^ in the photostationary CO-bound spectra is due to the loss of the non-hydrogen bonded heme Fe–His38⋯Gly359 conformer. In the ferrous heme *a*_3_ of oxidases the stretching frequency of the proximal histidine–iron mode *ν*_Fe–His_ falls at 193–214 cm^−1^ suggesting that the weak Fe–His bond may cause a strengthening of the Fe–CO bond.^9,10^ The reported Fe–CO and C–O frequencies of heme *a*_3_ indicate the presence of different active conformations in the binuclear center of *ba*_3_ preparations, which demonstrate the existence of conformational heterogeneity in the protein.^10–12^ Time-resolved step-scan FTIR spectroscopy has been utilized extensively in the ns–ms time range to probe the dynamics of *ba*_3_ and oxidoreductase.^13–22^ The presence of both protonated and deprotonated forms of the ring A of heme *a*_3_ propionate and the deprotonated form of Asp372 has been determined by time-resolved Fourier transform infrared spectroscopy on the *ba*_3_–CO complex.^19^ Based on recent Molecular Dynamics (MD) results, it was demonstrated that water molecules inside the protein are involved in the proton pumping activity as proton carriers and the highly conserved water molecule that lies between the heme *a*_3_ propionates is capable of transferring its proton to propionate-A which affects the Fe oxidation state.^23^ The functional consequences of the heterogeneity to the catalytic activities of the enzyme remain to be explored.

Spin fluctuations in heme Fe(ii) are at the heart of heme-proteins functionality.^24,25^ Despite significant progress in the chemistry of Fe–heme proteins, the mechanisms that control spin state stabilization remain elusive. In *ba*_3_, one question asked, is how the structural reorganizations accompanying spin transition will influence the redox catalytic activity of the enzyme that takes place in the heme Fe *a*_3_–Cu_B_ binuclear center. It is well known that intermediate and/or LS species are characterized by higher reaction rates and smaller activation energies compared to the HS analogues.^24^ The difference is driven by a higher tendency of LS iron(ii) to be oxidized.

In this report, resonance Raman spectra taken with 441 nm excitation in the +60 °C to −120 °C temperature range were utilized to characterize the spin of the heme iron and allowed us to identify based on the frequency shifts of the spin-state marker bands of heme *a*_3_, a reversible transition of the heme *a*_3_ Fe with a spin transition temperature *T*_1/2_ = −60 °C. Resonance Raman with excitation wavelength at 441 nm is in resonance with the Soret band that arise from a π–π* of the hemes and is sensitive to the charge, spin and ligation state of the heme Fe. An analysis of the temperature-dependent spectra can provide information on the dynamic properties of the protein in the moiety of the heme Fe. The temperature-dependent spin state transition that we observed in *ba*_3_ is best explained in terms of the displacement of the heme iron along the heme normal as a consequence of the Fe–Np repulsion. This way, the ligand field strength parameter will increase, shifting the transition towards a low-spin state. The transition we observed has been rarely reported in heme Fe proteins and is insensitive to H_2_O/D_2_O and H_2_O^18^ exchanges indicating that the internal perturbations including hydrogen-bonding and hydrophobic contacts,^20–22^ although can influence the energy splitting to create the spin transition, do not affect the frequency shifts of the spin marker bands.

## Experimental

Cytochrome *ba*_3_ was isolated from *Thermus thermophilus* HB8 cells according to previously published procedures.^1^ The *ba*_3_ samples were placed in a desired buffer 0.1 M pH/pD 7.0, HEPES [4-(2-hydroxyethyl)piperazine-1-ethanesulfonic acid]. The buffer prepared in D_2_O was measured assuming pD = pH (observed) + 0.4. The concentration of the samples was determined optically, using *ε*_416,ox_ = 152 mM^−1^ cm^−1^ and was ∼1.0 mM. H_2_^18^O and D_2_^16^O were purchased from Sigma-Aldrich. The dithionite reduced *ba*_3_ samples were placed in an anaerobic temperature controlled FTIR600 cell purchased by Linkam Scientific Instruments Limited. The desired temperature was achieved using the T95 and LN95 temperature controllers along with the use of liquid N_2_ and controlled by the LinkSYS software, all of the above were also purchased by Linkam Scientific Instruments Limited. The resonance Raman spectra were collected by the Synapse CCD detector purchased by HORIBA Jobin Yvon attached to iHR550 Raman Imaging spectrometer (Horiba Scientific) and the experiment parameters were controlled *via* SynerJY (HORIBA Jobin Yvon). A helium–cadmium 441 nm continuous wavelength laser beam (Kimmon Koha Co. Ltd) was used for the excitation of the *ba*_3_ samples. The accumulation time was 15 minutes for each measurement and approximately 15 measurements were collected and averaged to the final spectra. The temperature range of the experiments was from +60 °C to −120 °C. The first set of Raman measurements was collected when the sample was at *T* = 20 °C. The temperature of the sample was decreased to *T* = 10 °C and there was a waiting time of 15 min prior to the next collection of Raman data for the temperature of the sample to reach equilibrium. This procedure was repeated for every new temperature setting till the final temperature was −120 °C. Subsequently, the temperature of the sample was increased to room temperature and to *T* = +60 °C and for the final measurements was decreased again to −55 and −70 °C. Photoreduction was accomplished by explosion of the oxidized *ba*_3_ sample to 441 nm laser irradiation for 15 minutes.

## Results and discussion

**Fig. 1:**
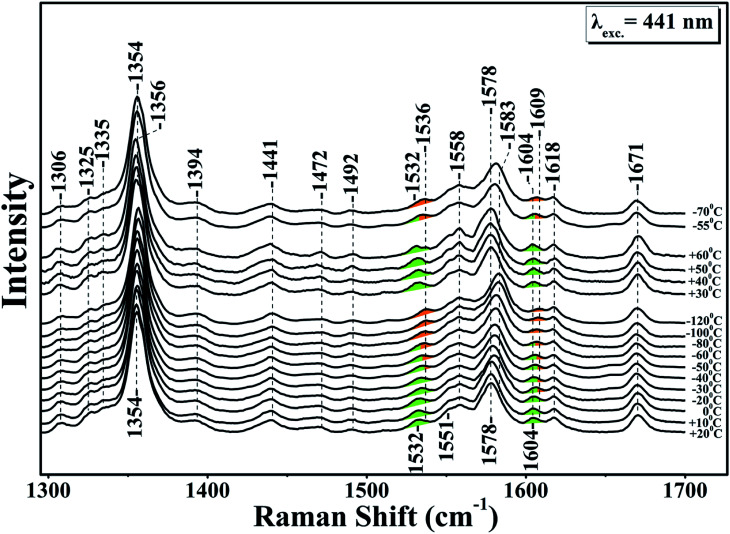
High frequency resonance Raman spectra of the reduced *ba*_3_ oxidase in D_2_^16^O, using sodium dithionite as a reducing agent. The excitation of Raman scattering was achieved using a helium–cadmium 441 nm continuous wave laser. The green colour indicates the position of the marker bands at room temperature and the orange colour the frequency progression at lower temperatures.

Fig. 1 shows the resonance Raman of the equilibrium reduced enzyme in the 60 to −120 °C temperature range. In the RR spectrum at *T* = 20 °C the oxidation state marker, *ν*_4_ with relatively small dependence on the spin state, is located at 1354 cm^−1^ show that the LS six-coordinate heme *b* and the HS five coordinate heme *a*_3_ are in the ferrous state.^28^ The HS sensitive *ν*_2_ band at 1578 cm^−1^ and the core-sensitive band *ν*_3_ at 1472 cm^−1^ clearly demonstrate that heme *a*_3_ is a five-coordinate, HS heme Fe. The *ν*_10_ HS marker band and the formyl of heme *a*_3_ are located at 1604 and 1671 cm^−1^, respectively. The nearly C_b_C_b_ stretching vibrations *ν*_11_ and *ν*_2_ are located at 1532 and 1579 cm^−1^, respectively. The *ν*_2_ of the HS six-coordinate heme *b* coincides with the *ν*_2_ of heme *a*_3_ at 1579 cm^−1^ whereas the *ν*(C=C) is observed at 1618 cm^−1^. No significant changes are observed in the RR spectra in the *T* = 20 to −30 °C range. In the *T* = −40 to −120 °C range the *ν*_4,_*ν*_11_, *ν*_2_ and *ν*_10_ vibrations are all shifted to higher frequencies. More specifically, in the *T* = −40 to −80 °C, *ν*_4_ shifts to 1356 cm^−1^ and the spin state marker bands *ν*_2_ and *ν*_10_ shift from their position observed in the *T* = 25 to −40 °C range. The *ν*_4_ band observed at 1356 cm^−1^ is ascribable to an in-phase combination of C_a_N and C_a_C_b_ stretch.^26^ This makes it sensitive to changes in the metal's oxidation and ligation states because both determine the extent of mixing between the d_π_ metal and the antibonding π* orbital of the heme macrocycle.^27^

**Fig. 2:**
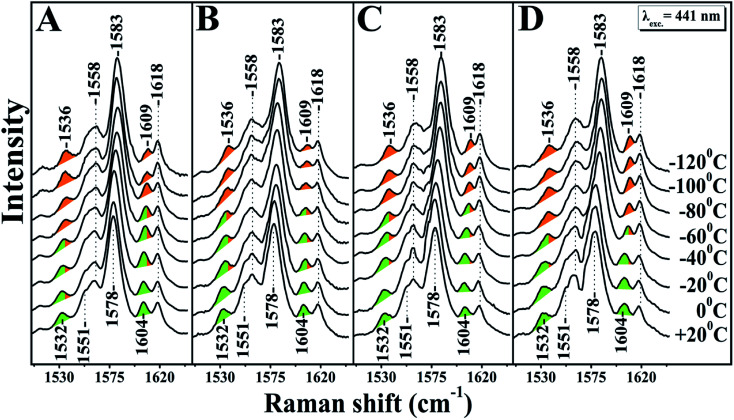
High frequency resonance Raman spectra of *ba*_3_ oxidase. Panel A represents the Raman spectra of the reduced *ba*_3_ oxidase using sodium dithionite as a reducing agent in H_2_^16^O. Panels B, C and D represent the Raman spectra of the photoreduced *ba*_3_ oxidase in D_2_^16^O, H_2_^16^O and H_2_^18^O, respectively. The excitation of Raman scattering was achieved using a helium–cadmium 441 nm continuous wave laser. The green colour indicates the position of the marker bands at room temperature and the orange colour the frequency progression at lower temperatures.

A large mixing yields significant electron backbonding to the π*-orbital, which shows considerable electron density in particular at the pyrrole nitrogens. After increasing the temperature from −120 to 60 °C the spin state marker bands and *ν*_4_ restore their initial frequencies observed at room temperature and subsequent decrease in temperature the spectra restore their previous negative temperatures at −55 and −70 °C. In the H_2_O/D_2_O/H_2_^18^O exchanged samples shown in panels A, B, C and D of Fig. 2, we have not seen any noticeable changes in the behaviour of the *ν*_4,_*ν*_11_, *ν*_2_ and *ν*_10_ marker bands in the *T* = −40 to −120 °C range. In the photoreduced samples, the heme *b* and *a*_3_ marker bands and their temperature behaviour are the same as those observed in the equilibrium reduced enzyme. Obviously, in the photoreduced *ba*_3_ the displacement (*Δ*) of the iron center from the mean plane of the heme unit is similar to that observed in the equilibrium reduced enzyme, thereby the electronic structure is not affected.

**Fig. 3:**
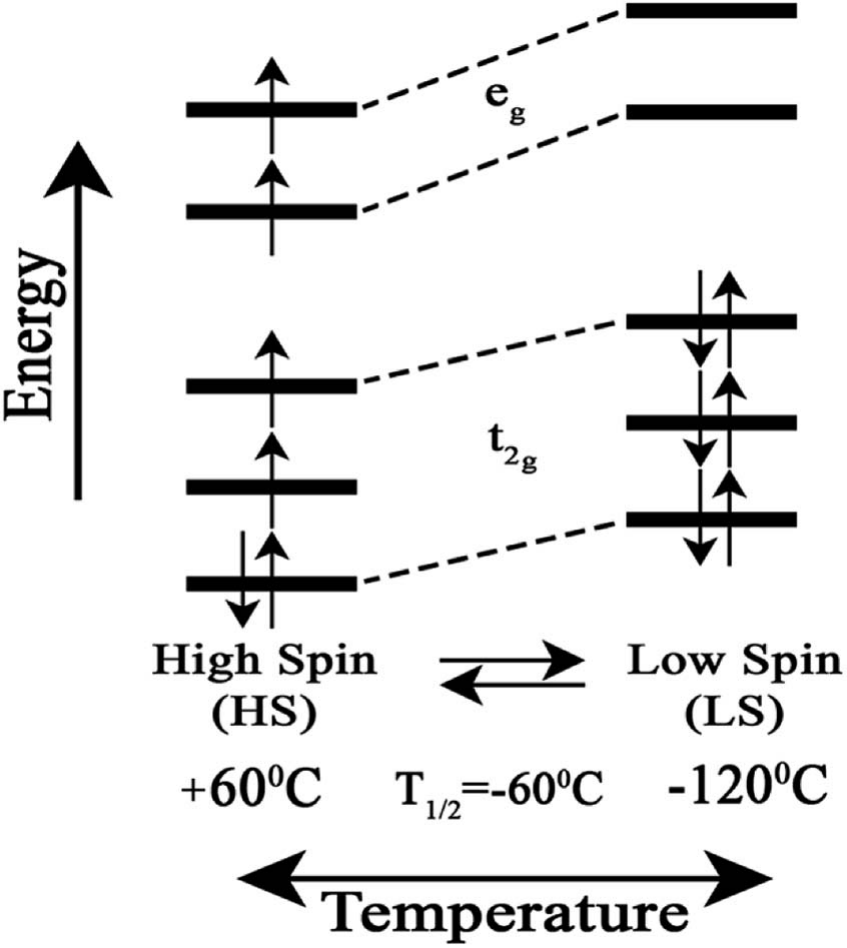
Schematic diagram for the reversible temperature-dependent high-spin to low-spin transition with *T*_1/2_ = −60 °C.

We attribute the changes we have observed in RR data as a function of temperature to a reversible spin transition. The temperature behaviour of the spin state marker bands observed in RR data indicates structural rearrangement in the heme *a*_3_ moiety. Fig. 3 depicts a schematic diagram for the reversible temperature-dependent high-spin to low-spin transition with *T*_1/2_ = −60 °C.

We suggest that the transition is accompanied by a displacement (*Δ*) of the heme iron along the heme normal as a consequence of the Fe–Np repulsion, resulting from the d_*x*^2^–*y*^2^_ molecular orbitals. Temperature can affect the metal displacement value *Δ*, thereby weakening the orbital overlap between 
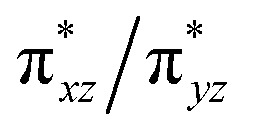
 and the e_g_ orbitals and consequently the energy splitting. In the photoreduced *ba*_3_ the similarity in *ν*_4_, *ν*_3_, *ν*_2_, *ν*_11_, *ν*_10_ and formyl vibration with the equilibrium deoxy form strongly suggest that the iron is at the same position in both forms of the enzyme. Therefore, in cytochrome *ba*_3_ we have established a protein tunable temperature dependent structural parameter which can be probed with respect to the possibility of being the link between the heme *a*_3_ site and protein structure.

Spin-transitions in Fe(ii) d^6^ electronic configuration systems with an N-based coordination sphere arranged are transition metal based molecular systems in a quasi-octahedral arrangement that can remain long in either one of two stable states – a low spin (LS) and a high spin (HS) state.^24^ For Fe(ii) complexes, one of the effects of the spin transition is that the formally antibonding e_g_ orbitals unpopulated in the low-spin (LS) state are populated in the high spin (HS) state and lengthening and weakening of the Fe–L bond lengths accompanies the LS → HS transition, with a consequent change in the volume of the complex and its vibrational characteristics. Transitions from one state to another can be induced by changing temperature or pressure or optically by irradiation. The light-induced excited spin state trapping phenomenon is of profound importance because of the possibility of optical switching. Thermal spin transition is entropy-driven from the populated HS state at high temperatures to the LS state which becomes populated at lower temperatures.^24^ The transition is possible when the zero-point energy difference between the HS and LS states Δ*H*^0^_HL_ is 0–1000 cm^−1^. An important parameter to characterize the temperature-driven spin transition is the transition temperature *T*_1/2_, which corresponds to the temperature at which the HS and LS states are equally populated. *T*_1/2_ has contributions from Δ*S*_HS_ and Δ*H*_HS_. The former contribution comes from the downshift of the vibrational frequencies under the spin-transition.

Strong cooperative interactions take place when a different transition temperature is observed by decreasing the temperature and by heating, when the reverse process takes place. The HS–LS electronic energy difference determines the relative positions of the minima of the potential energy surfaces obtained in the Born–Oppenheimer approximation for the LS and HS states, and thus, how long the system can remain within a particular state before thermal equilibrium is established. A temperature-dependent spin crossover in neuronal nitric oxide synthase bound with the heme-coordinating thioether inhibitors was reported, recently.^24^ It was reported that by lowering the temperature below 200 K, some thioether inhibitors show contracted Fe–S distance and switch from high to low spin similar to spin crossover phenomenon observed in many transition metal complexes. In addition, a SCO transition was recently reported to occur in Mb.^25^ Based on resonance Raman experiments it was demonstrated that the HS heme Fe–O–N=O complex is converted into a LS heme Fe–O–N=O/2-nitrovinyl that is reversibly switched. It was suggested that a structural rearrangement in the protein-binding pocket is responsible for the HS to LS spin-state change and the heme Fe–O–N=O/2-nitrovinyl species is accompanied by a displacement of the heme iron along the heme normal as a consequence of the Fe–Np repulsion.^25^

In *ba*_3_ we can exclude rearrangements in the distal site of the heme *a*_3_–Cu_B_ binuclear center by lowering the temperature. Heme *a*_3_ remains five coordinate in the *T* = +60 °C to −120 °C range. Therefore, the spin transition we have observed is not due to rearrangements, as it was observed in the Mb heme Fe–O–N=O complex, in the protein-binding pocket. Alternatively, if there is a contraction of the Fe–His384 bond, as it was observed for the Fe–S thioether distance in the case of Neuronal Nitric oxide Synthase, then a structural rearrangement in the proximal environment of heme *a*_3_ due to a change in the H-bonding interaction of His384 can also contribute to the spin transition through hydrogen-bonding interactions that affect the Fe–His384 bond length.

Regarding this aspect, in the proximal site steric repulsions between the pyrrole nitrogen atom and the δ- and ε-carbon atoms of the imidazole ring of the H- and non H-bonded of His384 can influence the electronic character. In this case there is a coupling of the a_2u_(π) porphyrin orbital to the d_*z*^2^_–σ Fe–N_ε_ antibonding orbital. A σ(Fe–NHis)–e_g_(π*) mixing, that populates the e_g_(π*) antibonding orbital can affect the *ν*_4_ and then correlation between *ν*_4_ and σ(Fe–His) is expected.^18,19^ Therefore, the frequency shift of the *ν*_4_ observed with *T*_1/2_ = −60 °C can be associated in addition to the Fe–Np repulsion, with the variation in the Fe–His distance.

## Conclusions

The five-coordinate HS heme *a*_3_ at room and high temperatures is reversibly converted to a LS five coordinated heme *a*_3_. The observed spin transition occurs with *T*_1/2_ = −60 °C and the LS heme *a*_3_ is expected to have higher tendency for oxidation of Fe(ii), as it has been observed in other LS Fe(ii) complexes. Cytochrome *ba*_3_ oxidase has a high oxygen affinity, expressed under elevated temperatures *T* = 47–85 °C and limited oxygen supply with unusual ligand binding properties of Cu_B_.^29^ Complete understanding of the thermodynamic and kinetic characterization of functional and physiologically relevant ligands, electron and proton pathways is a necessity for the elucidation of the adaptation mechanism. The behaviour of the cofactors involved in the peculiar ligand binding and electron transfer properties observed at room temperature with those at high and low temperatures will lead to a total decoding of the adaption mechanism. The spin transition we have observed it will be analyzed for chemical reactions of *ba*_3_ in order to derive a broader picture of the effect of spin state on the catalytic metal centers, in particular as photochemical and electrochemical activities may be very sensitive to the spin state of the heme *a*_3_.

## Conflicts of interest

There are no conflicts to declare.

## Supplementary Material
